# Infraslow closed-loop brain training for anxiety and depression (ISAD): a protocol for a randomized, double-blind, sham-controlled pilot trial in adult females with internalizing disorders

**DOI:** 10.1186/s13063-022-06863-z

**Published:** 2022-11-17

**Authors:** Tyson M. Perez, Paul Glue, Divya B. Adhia, Muhammad S. Navid, Jiaxu Zeng, Peter Dillingham, Mark Smith, Imran K. Niazi, Calvin K. Young, Dirk De Ridder

**Affiliations:** 1 Department of Surgical Sciences, University of Otago, Dunedin, New Zealand; 2 Department of Psychological Medicine, University of Otago, Dunedin, New Zealand; 3 Centre for Chiropractic Research, New Zealand College of Chiropractic, Auckland, New Zealand; 4 Donders Institute for Brain, Cognition and Behaviour, Radbout University Medical Center, Nijmegen, The Netherlands; 5 Department of Preventative & Social Medicine, Otago Medical School-Dunedin Campus, University of Otago, Dunedin, New Zealand; 6 Coastal People Southern Skies Centre of Research Excellence, Department of Mathematics & Statistics, University of Otago, Dunedin, New Zealand; 7 Neurofeedback Therapy Services of New York, New York, USA; 8 Department of Psychology, University of Otago, Dunedin, New Zealand

**Keywords:** Internalizing disorders, Generalized anxiety disorder, Major depressive disorder, Social anxiety disorder, EEG neurofeedback, Infraslow fluctuations, Triple network, Salience network, Default mode network, Central executive network, Randomized controlled trial

## Abstract

**Background:**

The core intrinsic connectivity networks (core-ICNs), encompassing the default-mode network (DMN), salience network (SN) and central executive network (CEN), have been shown to be dysfunctional in individuals with internalizing disorders (IDs, e.g. major depressive disorder, MDD; generalized anxiety disorder, GAD; social anxiety disorder, SOC). As such, source-localized, closed-loop brain training of electrophysiological signals, also known as standardized low-resolution electromagnetic tomography (sLORETA) neurofeedback (NFB), targeting key cortical nodes within these networks has the potential to reduce symptoms associated with IDs and restore normal core ICN function. We intend to conduct a randomized, double-blind (participant and assessor), sham-controlled, parallel-group (3-arm) trial of sLORETA infraslow (<0.1 Hz) fluctuation neurofeedback (sLORETA ISF-NFB) 3 times per week over 4 weeks in participants (*n*=60) with IDs. Our primary objectives will be to examine patient-reported outcomes (PROs) and neurophysiological measures to (1) compare the potential effects of sham ISF-NFB to either genuine 1-region ISF-NFB or genuine 2-region ISF-NFB, and (2) assess for potential associations between changes in PRO scores and modifications of electroencephalographic (EEG) activity/connectivity within/between the trained regions of interest (ROIs). As part of an exploratory analysis, we will investigate the effects of additional training sessions and the potential for the potentiation of the effects over time.

**Methods:**

We will randomly assign participants who meet the criteria for MDD, GAD, and/or SOC per the MINI (Mini International Neuropsychiatric Interview for DSM-5) to one of three groups: (1) 12 sessions of posterior cingulate cortex (PCC) ISF-NFB up-training (*n*=15), (2) 12 sessions of concurrent PCC ISF up-training and dorsal anterior cingulate cortex (dACC) ISF-NFB down-training (*n*=15), or (3) 6 sessions of yoked-sham training followed by 6 sessions genuine ISF-NFB (*n*=30). Transdiagnostic PROs (Hospital Anxiety and Depression Scale, HADS; Inventory of Depression and Anxiety Symptoms – Second Version, IDAS-II; Multidimensional Emotional Disorder Inventory, MEDI; Intolerance of Uncertainty Scale – Short Form, IUS-12; Repetitive Thinking Questionnaire, RTQ-10) as well as resting-state neurophysiological measures (full-band EEG and ECG) will be collected from all subjects during two baseline sessions (approximately 1 week apart) then at post 6 sessions, post 12 sessions, and follow-up (1 month later). We will employ Bayesian methods in R and advanced source-localisation software (i.e. exact low-resolution brain electromagnetic tomography; eLORETA) in our analysis.

**Discussion:**

This protocol will outline the rationale and research methodology for a clinical pilot trial of sLORETA ISF-NFB targeting key nodes within the core-ICNs in a female ID population with the primary aims being to assess its potential efficacy via transdiagnostic PROs and relevant neurophysiological measures.

**Trial registration:**

Our study was prospectively registered with the Australia New Zealand Clinical Trials Registry (ANZCTR; Trial ID: ACTRN12619001428156). Registered on October 15, 2019.

**Supplementary Information:**

The online version contains supplementary material available at 10.1186/s13063-022-06863-z.

## Introduction

### Background and rationale

Mental disorders are one of the most common causes of morbidity and mortality worldwide [1] with rates markedly increasing in recent years [2–6]. Here in New Zealand, it is estimated that one in five people is suffering from mental illness at any given time with a majority likely to experience at least one episode at some point in their lifetime [7]. Internalizing disorders (IDs, e.g. generalized anxiety disorder, GAD; social anxiety disorder, SOC; major depressive disorder, MDD; posttraumatic stress disorder, PTSD) are the most prevalent psychopathologies experienced worldwide [1, 8–11] and can be broadly characterized by a proclivity to direct distress inwardly [12–16]. Notably, IDs are highly comorbid [17–19] with females [20–23] and young people (i.e. <65 years) [8, 10, 17–19, 22, 24–29] disproportionately affected.

In recent years, neuropsychiatric research is pointing to transdiagnostic, neurobiological aberrations specifically involving the so-called core intrinsic connectivity networks (c-ICNs) which include the default mode network (DMN), central executive network (CEN) and salience network (SN) [30–33]. Briefly, the DMN is anchored in the posterior cingulate cortex (PCC) and medial prefrontal cortex (mPFC), and putatively subserves internally directed thought [34, 35]. The CEN, anchored in the dorsolateral prefrontal cortex (dlPFC) and posterior parietal cortices (PPC), is associated with executive functioning [36–42]. Lastly, the SN, anchored in the anterior insula (aINS) and dorsal anterior cingulate cortex (dACC), is believed to be important for the detection of salient stimuli and switching between the other c-ICNs [43, 44]. Additionally, the c-ICNs have also been associated with autonomic nervous system (ANS) modulation [45–50] possibly helping to explain the ANS dysfunction consistently reported across psychopathologies [51, 52].

In 2011, the converging neurobiological evidence led Menon and colleagues to propose a unifying theory of psychopathology termed the ‘triple network model’ [31, 53–55]. The central tenet of this theory is that the sensorial, cognitive, affective, and behavioural dysfunctions associated with mental illnesses are the result of disruptions within and between the c-ICNs. Since its inception, support for this model has been rapidly mounting within the ID-domain (e.g. [56–59]). Notably, to our knowledge, our lab was the first to validate this model with (source-space) electroencephalography (EEG) [60].

EEG non-invasively tracks and records electrophysiological signals generated by the brain [34–36]. Although traditionally used to assess activity in sensor-space, modern source-space algorithms (e.g. low-resolution brain electromagnetic tomography, LORETA [61, 62]) now allow accurate estimations of the regions (i.e. nodes) responsible for generating the scalp-recorded electrophysiological signals. Further, although standard clinical EEGs typically limit the recording bandwidth to traditional frequency bands (i.e. delta ~1-4 Hz, theta ~4-8 Hz, alpha ~8–12 Hz, beta ~12–30 Hz, gamma >30 Hz), acquisition and analyses of frequencies at the low end of the spectrum, commonly termed electrophysiological infraslow fluctuations (eISFs; <0.1 Hz), are now possible [63]. Russian scientists discovered eISFs over half a century ago, first in rabbits [64, 65] and shortly thereafter in humans [66] but, due in large part to technological challenges, they have received little interest from the scientific and clinicical communities until recently [63, 67–70]. Putatively engendered by a combination of neuronal and glial currents [63, 68, 71–76], eISFs have been shown in both cortical [65, 66, 77] and subcortical [76, 78–82] tissues and are believed to coordinate large-scale ICN organization and long-range information exchange [68, 83–91]. As such, treatments specifically targeting eISFs within core nodes of the triple network may address c-ICN dysfunction and offer clinical utility in the treatment of IDs.

Although traditional frontline therapies (i.e. pharmacotherapy and psychotherapy) are effective for many, they offer numerous shortcomings including high failure rates [92–98], lack of access [22, 99–102], and marked adverse side-effects [52, 99, 100, 103–105]. Closed-loop brain training of electrophysiological (EEG) signals, also known as EEG-neurofeedback (EEG-NFB), is a non-invasive therapy aimed at modulating brain function by teaching individuals, via associative learning (e.g. operant conditioning), to self-regulate their brain function via auditory, visual, and/or tactile feedback [106]. Intriguingly, EEG-NFB’s impact on the brain may intensify following the cessation of therapy putatively due to treatment-induced neuroplasticity; however, a general lack of extended follow-up and failure to assess for the emergence of delayed treatment effects is common in the literature [107]. That said, sceptics assert that comparable clinical improvements in both experimental and control groups in randomized, double-blind, sham-controlled trials suggest that EEG-NFB's efficacy rests entirely on ‘non-specific’ psychosocial factors (i.e. expectations, motivation, demand characteristics, context) [108–119]. However, proponents contend that evidence of differential EEG-learning (i.e. greater change in the targeted electrophysiological variable(s) and/or region(s)-of-interest (ROIs) in the genuine versus sham groups), considered by many to be essential for a valid evaluation of EEG-NFB’s specificity [120–126], was noticeably absent in the trials presented as evidence for wholly non-specific effects [127, 128]. That said, assessments of differential EEG-learning are complicated by a lack of standardized criteria for the determination of learning (or a lack thereof) [129]. In any case, EEG-NFB has shown promising clinical effects in a wide various of conditions [130–135] including IDs [102, 136–155]. Further, clinicians have reported success in ID populations using sensor-space EEG-NFB targeting eISFs (ISF-NFB) [156–158]. Advanced source-localization (i.e. standardized LORETA, sLORETA [62]) combined with ISF-NFB is a novel introduction to the field that has been shown by our research group in a feasibility trial on obese females to improve sleep and wellbeing with minimal side-effects [159, 160].

To our knowledge, this is the first randomized, double-blind, sham-controlled trial examining the potential effects of source-space ISF-NFB in an ID population. Furthermore, our relatively novel transdiagnostic approach heeds recent calls for a more pragmatic, ecologically valid clinical research [161–171]. As detailed below, our primary objectives will be to examine patient-reported outcomes (PROs) and neurophysiological measures to (1) compare the potential effects of sham ISF-NFB to either genuine 1-region ISF-NFB or genuine 2-region ISF-NFB and (2) assess for potential associations between changes in PRO scores and modifications of EEG activity/connectivity within/between the trained ROIs.

### Study objectives

#### Primary research questions

##### Objective 1

To compare the potential effectiveness of genuine ISF-NFB versus sham ISF-NFB for treating IDs in a female population. We will assess differences between sham ISF-NFB (sham) and single-region ISF-NFB (ISF1) or multi-region ISF-NFB (ISF2) in PROs and neurophysiological measures (i.e. EEG and HRV) after 6 training sessions (post 6 sessions). We hypothesize that all groups will show clinical improvements via non-specific (e.g. placebo) effects; however, ISF1 and ISF2 groups will demonstrate additional improvements due to specific effects (i.e. effects specific to the modulation of the trained ROIs).

##### Objective 2

To assess whether there is a potential relationship between changes in PROs and EEG variables post 6 sessions. Specifically, we are interested in whether there is evidence for an association between changes in the primary PRO (i.e. Hospital Anxiety & Depression Scale, HADS) scores and targeted ROI activity and connectivity.

#### Secondary research questions

##### Objective 3

To assess the potential clinical effects of an additional 6 treatment sessions (i.e. post-6 to post-12 sessions), we will examine changes in PROs and neurophysiological measures amongst the ISF1 and ISF2 groups. We hypothesize that additional sessions will provide additional benefits for both treatment groups.

##### Objective 4

To explore the potential for increased medium-term effects from ISF-NFB treatment at 1-month follow-up, we will examine changes in PROs and neurophysiological measures amongst the ISF1 and ISF2 groups.

##### Objective 5

To explore whether any potential associations observed in Objective 2 can be extended to post 12 sessions and 1-month follow-up amongst the ISF1 and ISF2 groups.

### Trial design

This study is a randomized, double-blind (participants and assessors), yoked-sham controlled (playbacks of genuine ISF-NFB sessions from another female ID participant at the same training stage), parallel-group (3-arm = sham, ISF-1, ISF-2), superiority, pilot trial with an allocation ratio of 2:1:1 (sham:ISF1:ISF2).

## Methods: participants, interventions, and outcomes

### Study population and setting

Our target population is adult females meeting the DSM-5 criteria for one or more IDs of interest (i.e. GAD, SOC and/or MDD). Our trial will recruit all participants from the community in and around Dunedin, New Zealand and be undertaken at the Departments of Surgical Sciences and Psychological Medicine, University of Otago, Dunedin, New Zealand.

### Eligibility criteria

Inclusion criteria:

1. Able to give informed consent
2. Adult between 18 and 64 years old
3. Biological female
4. Meets the DSM-5 criteria for one or more of the following current diagnoses:
  - GAD
  - SOC
  - MDD
5. Never undergone EEG-NFB therapy

Exclusion criteria:

1. Starting new medications or altering dosages of existing medications <4 weeks prior to their 1^st^ baseline session or at any time during the trial.
2. Currently taking short-acting benzodiazepines (i.e. midazolam, triazolam)
3. Undergoing intensive psychotherapy (e.g. cognitive behavioural therapy)
4. Any externalizing disorder (e.g. antisocial personality disorder, alcohol/substance abuse disorder)
5. Any thought disorder (e.g. mania, bipolar disorder)
6. Any Neurological disorder (e.g. epilepsy)
7. Deemed to be at high-risk of suicide per the Columbia-Suicide Severity Rating Scale (C-SSRS – Screen Version)
8. Pregnant females
9. Pacemaker
10. Post-concussion syndrome

Drop-out criteria:

1. Refusal to participate
2. Misses >1 intervention session

### Additional consent provisions for collection and use of participant data and biological specimens

Not applicable: no biological specimens will be collected, and all data is to be used solely in accordance with this trial.

### Explanation for the choice of comparators

Our choice of sham-controls allows us to elucidate any potential specific (e.g. non-placebo) effects and addresses widespread concerns of generally weak methodological designs in NFB trials [102, 172–174].

### Recruitment

To reach the widest possible audience, ID participants will be recruited via both posters placed around the city and targeted Facebook ads with an invitation to participate in a University of Otago mental health study. Advertisements will direct potential participants to a webpage that will describe the trial and invite those interested to complete a short online form which will query basic information including first name, age, date of birth, sex, ethnicity, education level, handedness, mental health history, pregnancy status, presence of electronic implants (i.e. pacemakers), email address, and phone number. Individuals who complete the online form and meet the basic qualifications will be contacted via email and asked to attend an in-person mental health interview at the University of Otago Hospital, Dunedin, New Zealand. Those that agree will be provided directions to the lab and a digital copy of the 7-page participant information sheet (PIS). A reminder text will be sent to potential participants on the day of their interview. Recruitment will continue until our target sample sizes are met and is expected to take 18–24 months. To help foster our recruitment efforts, all participants who complete the study will receive a $40 supermarket voucher as reimbursement for any parking expenses.

### Who will take informed consent?

At the initial meeting, a male doctoral/PhD student will (1) provide each potential participant with a paper copy of the participant information sheet written in English, (2) query if they have read and understood the document, (3) ask if they have any questions about the trial, and (4) request written informed consent from individuals willing to participate in the study. Participants will be informed that they may withdraw at any time without giving a reason and that all data collected up to the point of withdrawal may be used in the final analyses.

### Screening

Following the attainment of informed consent, a trained male doctoral/PhD student conduct the Mini-International Neuropsychiatric Interview (MINI; English version 7.0.2 for DSM-5) [175]. The MINI is a brief structured diagnostic interview, shown to be both valid and reliable, used to assess the 17 most common psychiatric disorders including MDD, suicidality, bipolar, panic disorder, agoraphobia, SOC, obsessive-compulsive disorder, PTSD, alcohol use disorder, substance use disorder, psychoses, anorexia, bulimia, binge-eating disorder, GAD, and anti-social personality disorder [176, 177]. In the event that the interviewer suspects that the interviewee is at high-risk for suicide per the MINI, he will screen using the Columbia-Suicide Severity Rating Scale (C-SSRS – Screen Version [178]) with affirmative answers to questions 4, 5 and/or 6b initiating immediate referral to Emergency Psychiatric Services. Those meeting the eligibility criteria will be enrolled into the study, have their anthropometric (i.e. height and weight) measurements taken, and be scheduled for their baseline assessments. Participants will also be familiarized with the study equipment, procedures, and personnel.

### Baseline assessments

Baseline assessments will take place on two separate occasions approximately 1-week apart with baseline #2 values used as reference. Duplicate baseline assessments will be performed to mitigate the influence of certain non-specific effects (i.e. regression to the mean [179] and elevation bias [180]) that may confound clinical trials. All assessment sessions for a given subject will take place at approximately the same time of day and be led by a female research assistant. Prior to each assessment session, participants will be asked to abstain from (1) food and water for 2 h, (2) smoking/vaping for 8 h, and (3) strenuous exercise, alcohol, caffeine and over-the-counter medication for 24 h. A reminder email and text will be sent to each participant one day prior to and on the day of the assessment sessions, respectively. Adherence to lifestyle restrictions will be queried at the beginning of each session with any breaches recorded. In cases of serious breaches (e.g. consumption of alcohol in the prior 24 h), assessment sessions will be rescheduled. In addition, the subject’s previous night’s sleep duration will be documented, and they will be asked to use the toilet immediately prior to testing to ensure an empty bladder. Together, these standardization procedures are in line with current recommendations for neurophysiological data collection [181–183] and will help to control for variability in neurophysiological output stemming from important factors like circadian rhythms [184, 185], gastric distention [182, 186, 187], hydration levels [182, 188], bladder distention [182, 189], caffeine [190, 191], nicotine [192, 193] and alcohol [194, 195].

All PROs (English versions) will be re-created in digital form via Qualtrics [196] which will allow participants to complete them using an iPad during their EEG set-up. To prevent missing data, a visual alert will be generated if any queries on a given form have missing responses. Research has indicated the electronic data collection increases the speed, accuracy, and user acceptability of the process [197–199]. The estimated total time to complete the battery of PROs is 20 min. The order of PRO administration will be standardized and based on questionnaire length (i.e. IDAS-II > MEDI > HADS > IUS-12 > RTQ-10).

Following completion of their PROs, neurophysiological data will be collected from each participant using Compumedics Neuroscan SynAmps RT 64-channel amplifier (DC mode, input impedance >10 GΩ, 24-bit analogue-to-digital resolution, common mode rejection >110 dB) using a continuous sampling rate of 1000 Hz. Recordings will take place in a quiet, cool (~15°C), dimly lit room as participants are seated upright in a comfortable chair with their eyes closed. We chose the eyes-closed condition because it has been reported to improve EEG reliability [200, 201]. The 10.5 min resting-state full-band EEGs (fb-EEGs) will use high-density (64-channel) silicone Quik-Cap Hydro Net caps with Ag/AgCl electrode placements corresponding to the international extended 10/20 system. The ground electrode is positioned at AFz with the reference electrode midway between Cz and CPz. Electrooculography (EOG) will track vertical and horizontal eye movements. The cap is soaked in a saline solution at least 30 min prior to application and all electrode impedances will be kept below 10 kΩ. To help reduce impedances, subjects will be asked to arrive with non-braided, dry, clean (i.e. no conditioner, gels, pastes, or sprays) hair. Concurrent with the resting-state EEG, a spontaneous breathing standard limb lead (lead-II) electrocardiogram (ECG) using Ag/AgCl electrodes will be performed. Following, a 10 min metronome paced breathing (12 breaths per minute) ECG with a 1:1 inspiratory/expiratory (I/E) ratio (i.e. 2.5-s inhalation/2.5-s exhalation) was collected. During pacing, participants will be instructed to breathe through their nose at normal depth (i.e. no deep breathing).

### Randomization

Following baseline #2 measurements, participants were randomized to one of 3 arms: (1) yoked-sham, (2) ISF1 = PCC up-training, or (3) ISF2 = concurrent PCC up-training and dACC down-training. Sham participants were offered active ISF-NFB upon their completion of the trial, thereby minimizing the potential of sham trial-associated participation barriers [202] as well as addressing any potential ethical concerns of sham-only allocations.

### Sequence generation

The randomization scheme will be generated by using the Web site Randomization.com [203] by a lab member from our group with no direct contact with the participants. This tool is a valid randomization program utilized by clinical trial researchers. Block randomization with random block sizes and a 2:1:1 (sham: ISF1: ISF2) allocation will be utilized.

### Concealment mechanism

Randomization sequences were kept in the central office in sequentially numbered, sealed, opaque envelopes prepared by the lab member who generated the randomization scheme. To ensure concealment, the block sizes will be known only by this lab member and not be disclosed to any of the researchers who have contact with the participants.

### Implementation (enrolment and assignment)

TMP is responsible for participant enrolment and will assign participants to interventions following baseline assessments and upon arrival at their first ISF-NFB session.

### Who will be blinded?

This is a double-blind study whereby participants and raters will be unaware of group assignments. The ISF-NFB trainer will not be blinded. To improve participant blinding, all aspects of sham sessions will be identical to active sessions including the live recording of sham participants’ EEGs along with real-time artefact alerts. Blinding integrity will be assessed post 6 sessions via a brief electronic questionnaire whereby participants will be queried as to (1) their perceived group allocation, (2) confidence in their answer to question 1 on a scale of 0–100%, (3) reason for their answer to question 1, and (4) if their group assignment was revealed to them in any way.

### Procedure for unblinding if needed

Treatment assignment will be disclosed to trial participants only upon their completion of the study.

### Intervention descriptions

Training sessions will commence within 1-week after baseline #2 assessments. To help reduce impedances, subjects were asked to arrive with non-braided, dry, clean (i.e. no conditioner, gels, pastes, or sprays) hair. Participants will attend three 30-min sessions per week, every other day, over 4 consecutive weeks (12 sessions in total). 19-channel sLORETA ISF-NFB training will be performed using a DC coupled amplifier produced by Brainmaster Inc. and the BrainAvatar software (version 4.7.5.844) in a quiet, cool (~15°C), dimly lit room by an unblinded male researcher with >2 years of experience in the administration of NFB. Participants will be seated in a comfortable chair and an appropriately sized Comby EEG cap will be placed on the participant’s head. Using a blunt need and syringe, the scalp will be mildly abraded prior to the application of an electrolyte gel beneath each electrode. It should be noted that the purpose of the cool room and scalp abrasion is to mitigate contamination of the EEG signal by electrodermal (i.e. sweat gland) potentials which are known to mimic brain-derived eISFs [63, 69]. Nineteen-channel EEGs will be recorded with the silver/silver chloride (Ag/AgCl) electrodes positioned according to the International 10–20 system (i.e. Fp1, Fp2, F3, F4, C3, C4, P3, P4, O1, O2, F7, F8, T3, T4, T5, T6, Fz, Cz, Pz) using a linked mastoids reference and a ground electrode positioned centrally between F3, Fp1, Fz and Fpz. The impedances of the active electrodes will be kept below 10 kΩ and a 50 Hz notch filter will be set.

Immediately prior to each training period, a demonstration of motion artefact alerts will be performed with instructions to avoid eye/head/face movements to minimize this non-rewarding feedback. Participants will then be instructed to close their eyes, relax, stay awake, and listen to the sound being played. They will be informed that the sound they hear reflects that they are doing well. Notably, no explicit strategies or instructions were given as, with few exceptions [204], implicit strategies have been shown to produce better outcomes [205–210].

**Fig. 1:**
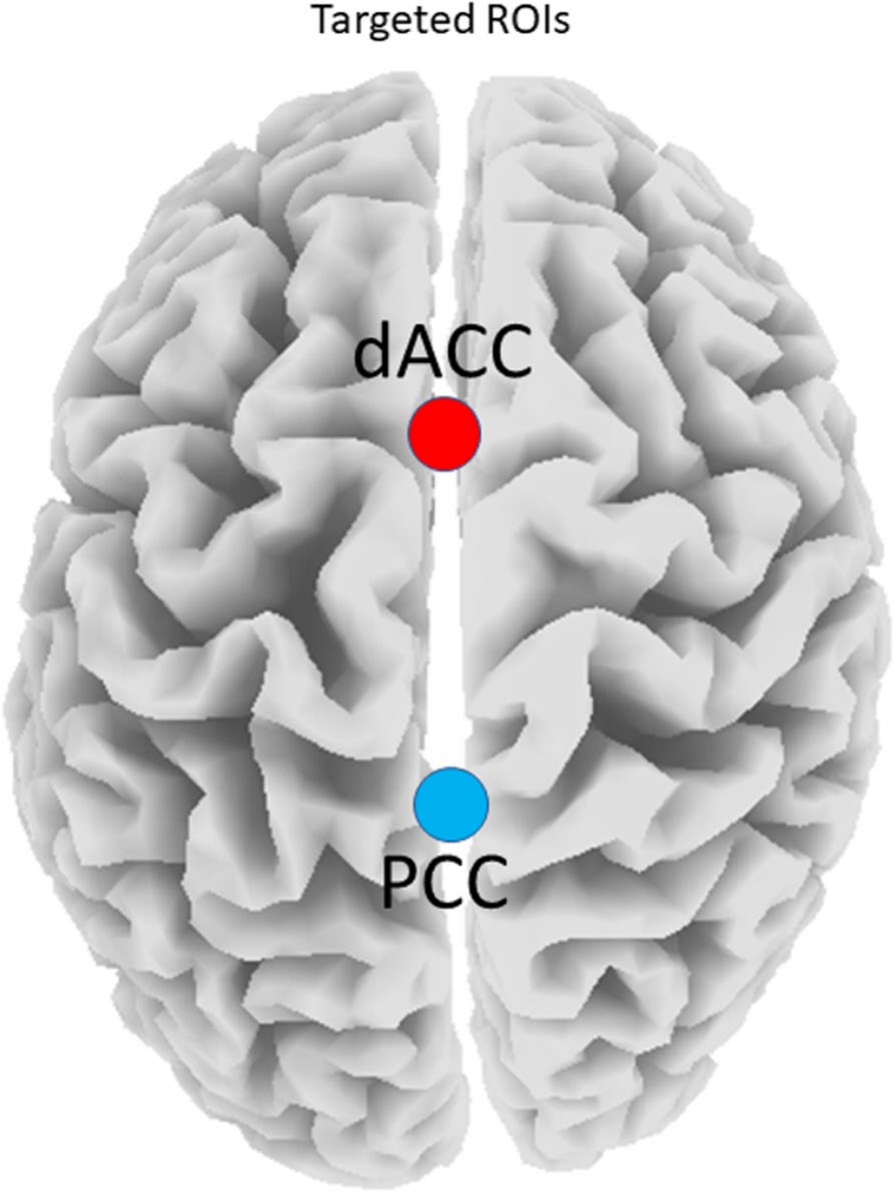
Targeted regions-of-interest (ROIs). Abbreviations: dACC, dorsal anterior cingulate cortex; PCC, posterior cingulate cortex. Note: red dot indicates salience network (SN), and blue dot indicates the default mode network (DMN) node

Continuous, real-time auditory feedback will be used for reinforcement and produced within 30 ms of the subject’s eISFs (0.0–0.1 Hz) within the pre-defined ROIs (i.e. dACC and/or PCC; Fig. 1) surpassing the threshold(s). These ROIs were selected because, as outlined in the introduction, they are considered key cortical nodes within the core RSNs which are consistently found to be disrupted in ID populations. sLORETA permits the selection of any cortical region for feedback of the current density using voxels selected based on Montreal Neurological Institute (MNI) coordinates [211]. For a complete list of targeted voxels for this trial, see Additional files 1 and 2.

The reward threshold(s) will be manually adjusted in real-time to maintain a 60% ± 10% success rate. Manual, rather than automated, thresholding was chosen as it has been reported to lead to better EEG-learning [125, 173, 206, 208]. The yoked-sham sessions will be identical to active sessions, including live EEG recordings and real-time motion/EMG artefact alerts, however, the auditory rewards will derive from playbacks of genuine ISF-NFB sessions from another female ID participant at the same training stage recorded via free, open-source Audacity software [212] which uses the computer’s sound card as an audio to digital converter. Importantly, this type of control allows matching of rewards and performance across sham and genuine conditions, thereby controlling as much as possible the learning context and degree of motivation [213] while theoretically severing the operant conditioning aspect of EEG-NFB. Additionally, it has been reported that training effects are more robust when the clinician is present [214], therefore, irrespective of group assignment, the trainer will be present for the duration of all sessions. Further, the trainer will monitor the participants’ protocol adherence. A detailed description of the trial intervention using the Template for Intervention Description and Replication (TIDieR) [215] has been provided in Table 1.

**Table 1:** Template for Intervention Description and Replication (TIDieR)

Item number	Item	Description
1.	**Brief name** Provide the name or a phrase that describes the intervention.	Infraslow closed-loop brain training for Anxiety & Depression (ISAD)
2.	**Why** Describe any rationale, theory, or goal of the elements essential to the intervention.	Electrophysiological infraslow (<0.1 Hz) fluctuations (eISFs) are believed to coordinate and integrate information exchange within and between core-intrinsic connectivity networks (ICNs). Further, communication within and between core-ICNs has been found to be disrupted in internalizing disorder (ID) populations. We hypothesize that eISF neurofeedback (ISF-NFB) targeting key cortical nodes of these core-ICNs, may restore proper intra- and inter-network function and reduce ID-related symptoms.
3.	**What** Materials: Describe any physical or informational materials used in the intervention, including those provided to participants or used in intervention delivery or training of intervention providers. Provide information on where the materials can be accessed (e.g. online appendix, URL).	19-channel sLORETA ISF-NFB training will be performed using a DC coupled amplifier (Brainmaster Technologies Inc.), ASUS laptop computer (ASUSTek Computer Inc.; 64.0 GB RAM; Intel Core i7 processor) running BrainAvatar software (version 4.7.5.844), 24 (Ag/AgCl) electrode Comby EEG caps, blunt needle, 5ml syringe, and electrolyte gel (Electro-Cap International Inc.). Free, open-source software (Audacity.com) is used to record/playback all auditory rewards during active/sham sessions.
4.	Procedures: Describe each of the procedures, activities, and/or processes used in the intervention, including any enabling or support activities.	Subjects will be asked to arrive with non-braided, clean, dry hair. They will be seated in a comfortable chair with their eyes closed in a quiet, cool (~15°C), dimly lit room. An appropriately sized Comby EEG cap will be affixed to the head and, using a blunt need and syringe, the scalp will be mildly abraded just prior to the application of the electrolyte gel beneath each electrode. EEGs will be recorded with the Ag/AgCl electrodes positioned according to the International 10–20 system (i.e. Fp1, Fp2, F3, F4, C3, C4, P3, P4, O1, O2, F7, F8, T3, T4, T5, T6, Fz, Cz, Pz) using a linked mastoids reference and a ground electrode positioned centrally between, F3, Fp1, Fz and Fpz. Impedances will be kept below 10 kΩ. Immediately prior to each training period, a demonstration of motion/EMG artefact alerts will be provided with instructions to avoid eye/head/face movements to minimize this non-rewarding feedback. Participants will then be instructed to close their eyes, relax, stay awake, and listen to the sound being played. They will be informed that the sound they hear reflects that they are doing well. Continuous, real-time auditory feedback (organ tones) will be used for reinforcement when the subject’s ISFs surpass the threshold(s). The reward threshold will be manually adjusted in real-time to maintain a 60% ± 10% success rate. The yoked-sham sessions will be identical to active sessions, including live EEG recordings and real-time motion/EMG artefact alerts, however the auditory rewards will derive from playbacks of consecutive, pre-recorded sessions of another female with IDs. The trainer will remain present for the duration of all sessions to monitor the EEG.
5.	**Who provided** For each category of intervention provider (e.g. psychologist, nursing assistant), describe their expertise, background and any specific training given.	A non-blinded doctoral student with 2+ years of training and experience in the administration of NFB
6.	**How** Describe the modes of delivery (e.g. face-to-face or by some other mechanism, such as internet or telephone) of the intervention and whether it was provided individually or in a group.	sLORETA ISF-NFB sessions will be performed one-on-one and face-to-face
7.	**Where** Describe the type(s) of location(s) where the intervention occurred, including any necessary infrastructure or relevant features.	sLORETA ISF-NFB sessions will take place in the EEG lab of the Department of Psychological Medicine, University of Otago, Dunedin, New Zealand.
8.	**When and how much** Describe the number of times the intervention was delivered and over what period of time including the number of sessions, their schedule, and their duration, intensity or dose.	Participants will attend three 30-min sessions per week over 4 consecutive weeks (12 sessions in total).
9.	**Tailoring** If the intervention was planned to be personalized, titrated or adapted, then describe what, why, when, and how.	Auditory feedback during active sLORETA ISF-NFB sessions will be based on each person’s real-time EEG-derived cortical ISFs. Thresholds will be manually adjusted, as needed, to maintain the pre-specified feedback success rate (i.e. 60% ± 10%)
10.	**Modifications** If the intervention was modified during the study, describe the changes (what, why, when, and how).	Not applicable. This is a protocol.
11.	**How well** Planned: If intervention adherence or fidelity was assessed, describe how and by whom, and if any strategies were used to maintain or improve fidelity, describe them.	Protocol adherence will be monitored by the trainer. Attempts will be made to mitigate adherence issues via automated email and text message reminders sent on the day of each training session.
12.	Actual: If intervention adherence or fidelity was assessed, describe the extent to which the intervention was delivered as planned.	Not applicable. This is a protocol.

### Participant timeline

**Fig. 2:**
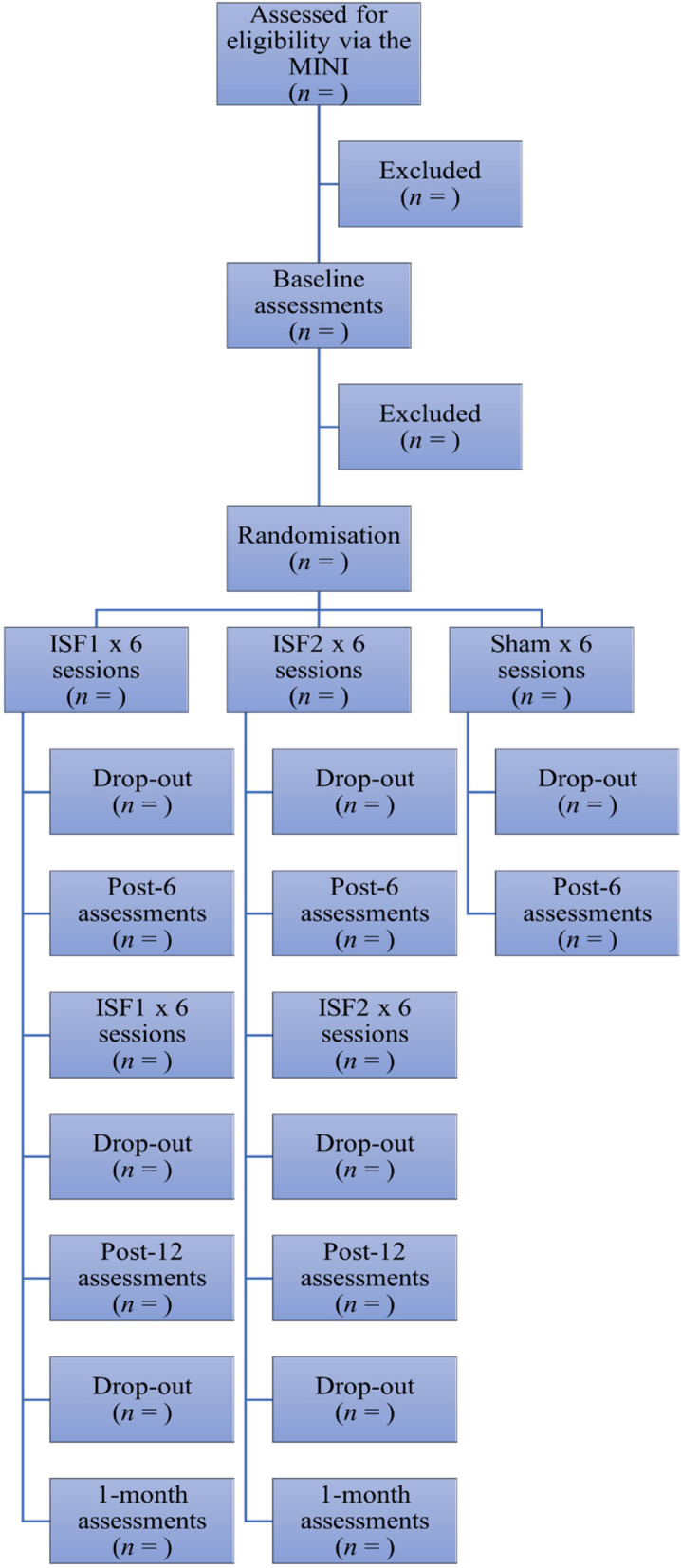
Flow chart of enrollment, assessments, and interventions. Abbreviations: MINI, Mini International Neuropsychiatric Interview; ISF1, 1-region Infraslow Neurofeedback; ISF2, 2-region Infraslow Neurofeedback

The trial period for each participant will be approximately 10 weeks and consist of one 30-min screening interview, two 60-min baseline assessments approximately 1 week apart, six 30-min genuine or sham ISF-NFB sessions (3× per week over 2 consecutive weeks) starting within 1 week after baseline #2, a 60-min post 6 sessions assessment, six 30-min genuine ISF-NFB sessions (3× per week over 2 consecutive weeks), a 60-min post 12 sessions assessment, and a 60 min 1-month follow-up assessment (Table 2 and Fig. 2). All post-treatment assessments and procedures will be identical to those performed at baseline.

**Table 2:** Schedule of enrollment, interventions, and assessments

	T0	T1	T2	Allocation	T3-8	T9	T10-15	T16	T17
Enrollment									
Eligibility screen (MINI)	✓								
Informed consent	✓								
Anthropometric measures	✓								
Assessments									
PROs		✓	✓			✓		✓	✓
EEG		✓	✓			✓		✓	✓
ECG		✓	✓			✓		✓	✓
DESS					✓		✓	✓	✓
Blinding integrity						✓			
Groups									
Active	✓	✓	✓	✓	✓	✓	✓	✓	✓
Sham	✓	✓	✓	✓	✓	✓			

*Abbreviations*: *MINI* Miniature International Neuropsychiatric Interview, *PROs* patient-reported outcomes, *EEG* electroencephalography, *ECG* electrocardiography, *T0* interview, *T1* baseline #1, *T2* baseline #2, *T3–8* neurofeedback sessions 1 through 6, *T9* post 6^th^ session assessments, *T10–15* neurofeedback sessions 7 through 12, *T16* post 12^th^ session assessments, *T17* 1-month follow-up assessments

### Criteria for discontinuing or modifying allocated interventions

Participants will be advised that they were able to withdraw at any time without giving a reason or may be withdrawn by the lead investigator if they (1) experience significant adverse effects that were deemed detrimental to their well-being or (2) are unable to adhere to protocol (e.g. missed >1 training session in either 6 session block, started or modified first-line therapies).

### Strategies to improve adherence to interventions

We will attempt to mitigate adherence issues via automated email and text message reminders sent on the day of each training session.

### Plans to promote participant retention and complete follow-up

Once enrolled, every reasonable effort will be made to follow participants throughout the entirety of the study period via ongoing email and text messaging correspondence. In the event of premature discontinuation of the study for any reason, participants will be made aware that all data collected up to the point of withdrawal may be used for analyses.

### Relevant concomitant care permitted or prohibited during the trial

Participants were asked to maintain any current first-line mental health therapies (e.g. pharmacotherapy) for the entire length of the trial period (i.e. baseline through follow-up). Any changes or introductions of first-line therapies (e.g. altered pharmacotherapy dosages, introduction of intensive psychotherapy) will render participants ineligible.

### Provisions for post-trial care

In the unlikely event of injury, participants will be eligible to apply for compensation from the Accident Compensation Corporation (ACC) of New Zealand just as they would be if they were injured in an accident at work or at home. Although there are private providers abroad (e.g. Asia, North America, and Europe), should this trial provide evidence of efficacy, there is currently no access to this therapy within New Zealand.

### Measurements

#### Primary outcomes: HADS, fb-EEG

The central importance of PROs in clinical trials has been emphasized by both international health regulatory agencies and patients [216, 217], therefore, the *primary* outcome of interest will be the HADS [218, 219]. Additionally, the importance targeted EEG-learning assessments in NFB trials has been emphasized by researchers [120–126], therefore activity (i.e. amplitude of the oscillations) and connectivity (i.e. coordinated amplitude and/or phase of the oscillations) changes within and between the targeted ROIs will also be of primary interest. Primary outcome measures will be collected at baseline, post 6 sessions, post 12 sessions, and 1-month follow-up.

##### Hospital anxiety depressions scale (HADS)

The HADS is a valid and reliable 14-item, trans-diagnostic PRO measure used to assess anxiety and depression severities [218]. Response options are on a 4-point scale (0–3) based on participants experiences over the past week with anxiety and depression subscale scores graded as follows: 0–7 = ‘normal’, 8–10 = ‘mild’, 11–14 = ‘moderate’, and 15–21 = ‘severe’ [220]. The HADS has been repeatedly shown to be a reliable and valid tool across a variety of settings [219, 221, 222]. There is some debate with respect to whether the HADS is best assessed via the total 14-item score [223–225] or two 7-item subscale (anxiety and depression) scores [219, 222, 225, 226]. For our trial, we are considering the anxiety (HADS-A) and depression (HADS-D) subscale scores separately. Importantly, the minimum clinically important difference (MCID) for the HADS subscales is estimated to be a reduction of 1.5 to 2 points [227–229].

##### Full-band EEG (fb-EEG)

**Fig. 3:**
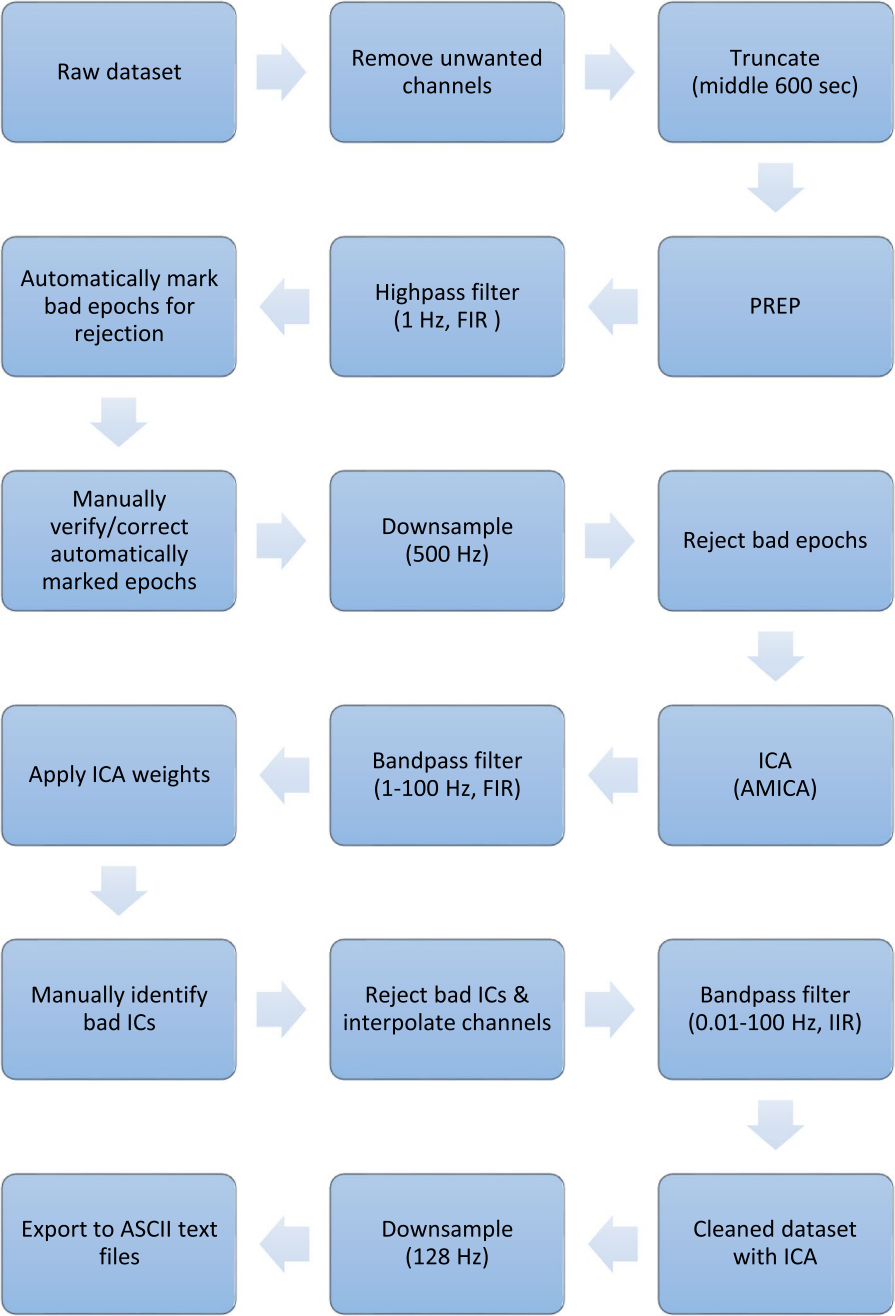
Overview of EEG pre-processing pipeline. FIR, finite impulse response; IIR, infinite impulse response; ICA, independent component analysis; AMICA, adaptive mixture ICA; ICs, independent components; Hz, Hertz

The use of full-band EEGs permits the non-invasive examination of the entire spectrum of brain frequencies from infraslow (<0.1 Hz) to gamma (>30 Hz) using scalp-recorded electrical signals acquired from direct current (DC) coupled amplifiers. Further, source-localization software can locate the probable generators (i.e. brain sources) responsible for the acquired electrical signals, thereby permitting us to assess activity (i.e. log-transformed current source density; log-CSD) and connectivity (i.e. lagged linear connectivity; lag-CON). EEG pre-processing will be performed offline using EEGLAB version 14.1.1 [230] and ERPLAB version 6.1.4 [231] running on MATLAB 2021a (The MathWorks, Inc., Natick, MA, USA.). Custom scripts developed in MATLAB utilizing EEGLAB, ERPLAB, and MATLAB functions will be used. The raw EEG will be imported into MATLAB using EEGLAB. Channel locations/coordinates will be determined via EEGLAB’s *Montreal Neurological Institute (MNI) coordinate file for BEM dipfit model* with the head centre optimized. Non-EEG (i.e. VEOG, HEOG, EKG, EMG, GSR) and four EEG (i.e. F11, FT11, F12, and FT12) channels will then be removed prior to manual co-registration used to match the coordinates of the 60 remaining channels to the realistic *Boundary Element Model (MNI)* head model*.* Of note, the four EEG channels selected for removal lack locations in the MNI coordinate file, thereby precluding subsequent pre-processing. The data will then be truncated to retain only the middle 600-s of the time-series, and the PREP pipeline version 0.55.1 [232] will be run to identify and interpolate bad channels, remove line noise, and robust average reference the data. This pipeline has been used previously for evoked potentials and resting-state EEG data [233, 234]. PREPed EEGs with >25% (i.e. >15) bad channels identified will not be considered for further analyses. For identification and marking of artefact-contaminated epochs, continuous PREPed EEGs will be 1 Hz high-pass filtered using a finite impulse response (FIR) filter implemented using EEGLAB’s pop_firwsord function (window = Kaiser 5.653, transition bandwidth = 1.5 Hz, max ripple = 0.001, order = 2416) and segmented into 1-s epochs. Epochs will automatically be marked as artefacts if containing one or more of the following characteristics: (i) absolute voltage exceeds 100 μV, (ii) peak-to-peak voltage exceeds 150 μV in any sliding window of 200 ms width with a step size of 100 ms, (iii) voltage greater than 100 μV resulting from a step-function with a sliding window 200 ms wide with a step size of 50 ms, (iv) sample-to-sample difference exceeding 50 μV, or (v) absolute voltage less than 1 μV for 150 ms (i.e. flat-lined data). Following this, manual verification/correction of epoch classifications (i.e. artefact and non-artefact) will be performed. Manually verified time-series with >50% (i.e. >5 min) artefact-contaminated epochs will be excluded from further analyses. For independent component analysis (ICA), the data will again be 1 Hz high-pass FIR filtered (window = Kaiser 5.653, transition bandwidth = 1.5 Hz, max ripple = 0.001, order = 2416), down-sampled to 500 Hz to reduce computation time, have noisy channels and artefact-contaminated epochs removed, and decomposed into maximally independent components (ICs) which are spatially fixed and temporally discrete [235] using adaptive mixture ICA (AMICA) [236]. AMICA was selected based on its superior performance when compared with other ICA algorithms [237]. The resulting ICA weights will be applied to 1–100 Hz band-pass FIR filtered (window = Kaiser 5.653, transition bandwidth = 1 Hz, max ripple = 0.001, order = 3624) data. We will use ICLabel [238] with manual verification to categorize the ICs as brain or other (i.e. muscle, eye, channel noise, line noise, or other) based on their spatial distribution (scalp topography), time course, spectrograms, event-related potential (ERP) images, and current dipole models using recommendations from Jung et al. [239], Chaumon et al. [240], and the website https://labeling.ucsd.edu/. Finally, bad ICs will be removed, noisy channels interpolated, and the data 0.01–100 Hz bandpass infinite impulse response (IIR) filtered (1^st^ order Butterworth) to give cleaned datasets. This IIR filter has been utilized in previous studies of eISFs [80, 241]. Finally, cleaned datasets with ICA will be downsampled to 128 Hz to reduce computation time and exported to ASCII text files for subsequent analyses. Figure 3 shows an overview of the EEG pre-processing pipeline.

**Fig. 4:**
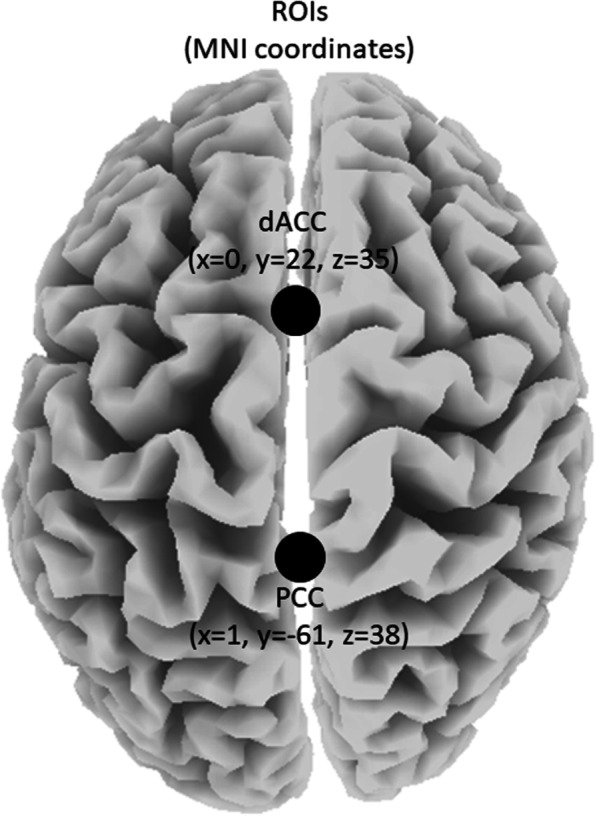
Regions-of-interest (ROIs) and their centre of mass coordinates. MNI, Montreal Neurological Institute; dACC, dorsal anterior cingulate cortex; PCC, posterior cingulate cortex

Via LORETA-KEY software (v20210701, freely available at http://www.uzh.ch/keyinst/loreta.htm), ASCII text files will be used as input to compute cross-spectral matrices for each participant for seven frequency bands (infraslow 0.01–0.1 Hz; slow 0.2–1.5 Hz; delta 2–3.5 Hz; theta 4–7.5 Hz; alpha 8–12 Hz; beta 12.5–30 Hz; gamma 30.5–44 Hz) utilizing fast Fourier transform (FFT). To allow for 2 complete cycles of the lowest frequency of interest (i.e. 0.01 Hz) and to obtain smooth power spectral density, EEGs will be segmented into 200-s epochs with Hanning (Hann) tapered windows applied. The cross-spectral matrices will then be averaged for each subject and used as input to exact LORETA (eLORETA) to compute whole-brain current source density (CSD; A/m^2^) without assuming a pre-defined number of active sources [242, 243]. Using the MNI-152 (Montreal Neurological Institute, Canada) template, eLORETA produces an inverse solution space consisting of 6239 cortical grey matter voxels at 5 mm resolution and has been shown to produce exact, zero-error localizations even in the presence of measurement and structured biological noise. eLORETA performs voxel-by-voxel between-condition comparisons of the CSD distribution. Statistical non-parametric mapping (SnPM) will be performed for each contrast using built-in voxel-wise randomization test (5000 permutations) to calculate the empirical probability distribution for the max-statistic (e.g. the maximum of a *t* or an *F* statistic) under the null hypothesis while correcting for multiple testing (i.e. for the collection of tests performed for all electrodes and/or voxels, and for all time samples and/or discrete frequencies). For each contrast, the voxel-level, two-tailed max-statistic was used as input to LORETA-KEY software to identify and visualize differences/changes in log-CSD for each of the seven frequency bands. Furthermore, for each condition, log-CSDs were averaged across all voxels within a 10-mm radius of the centre of mass MNI coordinates derived from previous literature [244] of the targeted ROIs (Fig. 4). This output was exported to Excel (version 2112) and analysed in R (version 4.0.5 [245];) to identify differences/changes in log-CSD between contrasts for each ROI in each frequency band. Next, functional connectivity (FC; i.e. lag-CON [242]) between the targeted ROIs was calculated for each group at each time point in LORETA-Key. Output was then exported to Excel and analysed in R to identify differences/changes between contrasts in each frequency band. Finally, as effective connectivity (EC) reflects directed functional connectivity, significant ROI pairs identified from FC analyses were selected for EC (i.e. Granger causality [246]) analyses. As with FC, EC was calculated in LORETA-Key, exported to Excel, and analysed in R.

#### Secondary outcomes: MEDI, IDAS-II, IUS-12, RTQ-10, HRV

The Multidimensional Emotional Disorder Inventory (MEDI) [247, 248], Inventory of Depression and Anxiety Symptoms – Second Version (IDAS-II) [249, 250], Intolerance of Uncertainty Scale – Short Form (IUS-12) [251], and Repetitive Thinking Questionnaire (RTQ-10) [252–254], and heart rate variability (HRV) will be considered *secondary* outcomes of interest which are also collected at baseline, post 6 sessions, post 12 sessions, and 1-month follow-up.

##### Multidimensional emotional disorder inventory (MEDI)

The MEDI, which has been validated on both clinical [247] and non-clinical [255] populations, is a 49-item, trans-diagnostic PRO measure that employs a response scale ranging from 0 (not characteristic of me/does not apply to me) to 8 (extremely characteristic of me/applies to me very much) to assess nine ID-related symptom domains, originally proposed by Brown and Barlow [256], including (1) Neurotic Temperament (5 items), (2) Positive Temperament (5 items), (3) Depression (5 items), (4) Autonomic Arousal (5 items), (5) Somatic Anxiety (5 items), (6) Intrusive Cognition (6 items), (7) Social Concerns (5 items), (8) Traumatic Re-experiencing (5 items), and (9) Avoidance (8 items). Although validated clinical severity thresholds for the MEDI total subscale scores are still lacking, the authors of this measure have suggested that average subscale scores >4 (or <4 for positive temperament), >6 (or <2 for positive temperament), and 7–8 (or 0–1 for positive temperament) may reflect moderate, severe, and extreme severities, respectively [257].

##### Inventory of depression and anxiety symptoms – second version (IDAS-II)

The IDAS-II is a valid and reliable 99-item, trans-diagnostic PRO measure that uses a response scale ranging from 1 (not at all) to 5 (extremely) to assess 19 current (past 2 weeks) ID-related symptom domains including general depression (20 items), dysphoria (10 items), lassitude (6 items), insomnia (6 items), suicidality (6 items), appetite loss (3 items), appetite gain (3 items), well-being (8 items), ill temper (5 items), mania (5 items), euphoria (5 items), panic (8 items), social anxiety (6 items), claustrophobia (5 items), traumatic intrusions (4 items), traumatic avoidance (4 items), checking (3 items), ordering (5 items), and cleaning (7 items) [249, 250]. Notably, in contrast to the other domains, the general depression domain is a composite of all 10 items from the dysphoria domain, as well as 2 items each from the suicidality, lassitude, insomnia, appetite loss and well-being domains. Recently, severity (mild, moderate, severe) thresholds have been introduced for 12 of the subscales including general depression, dysphoria, lassitude, insomnia, suicidality, appetite loss, appetite gain, well-being, ill-temper, panic, social anxiety, and traumatic intrusions [258].

##### Intolerance of uncertainty scale – Short form (IUS-12)

The IUS-12 is a valid and reliable 12-item, transdiagnostic PRO measure that assesses the degree to which an individual considers the possibility of a negative event occurring unacceptable, irrespective of its probability of occurrence [251, 259]. Whereas the original IUS-27 was GAD-specific, the IUS-12 has been distilled in order to measure the core intolerance of uncertainty construct [260]. The IUS-12 uses a response scale from 1 (“not at all characteristic of me”) to 5 (“entirely characteristic of me”) with total scores that can range from 12 to 60 [251]. Although some researchers have claimed that the IUS-12 is a unidimensional construct and recommend using only the IUS-12 total score [261–263], there has been considerable support for a two-factor IUS-12 structure: (1) a 7-item prospective IU scale related to action/approach-oriented strategies in order to increase certainty (e.g. seeking more information), and (2) a 5-item inhibitory IU associated with inaction/avoidance-oriented thoughts and behaviours (e.g. delayed decision-making) [251, 259, 264–271]. Intolerance of uncertainty (IU) is a common trait shared across the ID spectrum [271–274].

##### Repetitive thinking questionnaire (RTQ-10)

The RTQ-10 is a 10-item, trans-diagnostic PRO measure [253] distilled from three disorder-specific scales: 1) the MDD-associated Ruminative Responses Scale (RRS [275]), 2) the GAD-associated Penn State Worry Questionnaire (PSWQ [276]), and 3) the SOC-associated Post-Event Processing Questionnaire-Revised (PEPQ-R [277]). Participants are asked to rate the truthfulness of each statement with respect to their experience when they are “distressed or upset.” All items are rated along a 5-point scale: not at all true = 1, somewhat true = 3, or very true = 5 allowing total scores that can range from 10 (low levels of latent repetitive negative thinking) to 50 (extremely high levels of repetitive negative thinking). Repetitive negative thinking (i.e. rumination and worry) is a characteristic feature of IDs [278–280].

##### Heart rate variability (HRV)

Heart rate variability (HRV) is the phenomenon of cyclical beat-to-beat changes in the interbeat interval (i.e. RR interval), the dynamics of which can give insight into cardiac autonomic function [281]. ANS function is purportedly modulated by the core-ICNs and one of the most robust ANS disturbances found in IDs is cardiac dysautonomia in the form of reduced HRV [52, 96, 103, 280, 282–293]. This has significant clinical implications considering that cardiovascular disease is the leading cause of mortality in people with mental illness [52, 294, 295]. HRV is typically measured via the standard time-domain (i.e. standard deviation of normal-to-normal intervals, SDNN (ms), root mean square of successive differences between normal-to-normal intervals, RMSSD (ms)) and frequency-domain (i.e. LF-HRV = 0.04–0.15 Hz and HF-HRV = 0.15–0.4 Hz absolute power (ms^2^)) indices, however, non-linear analyses (e.g. Poincaré plot) are fast emerging as a way to characterize the complex, non-linear dynamics of cardiac-ANS interactions [296]. The raw ECG signals will be extracted in EEGLAB and saved as EDF (.edf) files. Using MATLAB 2021a and modifications to the open-source code for HRVTool version 1.07 (https://github.com/MarcusVollmer/HRV) [297], ECG time-series will be band-pass IIR filtered (10–35 Hz, 4^th^ order Butterworth) to remove signal drift and line noise, undergo automated annotation of R-peaks, and be interpolated using the shape-preserving piecewise cubic Hermite interpolating polynomial (Pchip) method to correct for artefacts (e.g. ectopic or missing beats). Pchip interpolation was chosen because appears to perform best across the spectrum of HRV metrics as it preserves the linear trend as well as the non-linear contributions in the R-R timeseries [298]. Using the HRVTool graphical user interface (GUI), Poincaré plots will also be examined to look for evidence of regularities (e.g. ‘comet’ shape) with those showing marked irregularities suggestive of cardiac arrhythmias (e.g. ‘fan’ shape) excluded from further analyses. Next, the first 10 s of ECG traces will be truncated to allow for signal stabilization followed by manual inspection/correction of the succeeding 5 min (i.e. 10–310 s) to ensure proper annotation of R-peaks generated from normal sinus rhythm with traces requiring >5% R-R interval interpolation excluded from further analyses. Finally, using the GUI’s R-R tachogram (time-series and spectrum), consecutive 60-s epochs (i.e. 1–5 min) from spontaneous breathing ECGs will be examined with those suggesting mean respiration rates outside of 0.15–0.4 Hz (i.e. 9–24 breaths per min) excluded from further analyses. Indices for both spontaneous and paced breathing conditions will be reported.

### Sample size

This is the first study examining the effects of sLORETA ISF-NFB in an ID population. Due to its novelty, there was no existing information around standard deviations for the measurements of interest. Therefore, no formal sample size or power calculations were made. Our group has previously carried out an sLORETA ISF-NFB trial in obese females [159]; however, this is a different population than the one in our trial and therefore not considered comparable for this study. Importantly, due to the pilot nature of this trial, only potential efficacy (or lack thereof) can be established via statistical analyses.

### Statistical considerations

#### Objective 1

Between-group (sham vs. ISF1 and ISF2) comparisons post-6 sessions will be performed for the PROs and neurophysiological measures. For this analysis, we will use LORETA-Key software and a Bayesian model with random effects to allow for baseline differences and non-specific temporal effects between participants. A sensitivity analysis will be carried out to compare this approach with an ANCOVA that includes a linear baseline adjustment.

#### Objective 2

Regression methods will be used to explore the relationship between changes in the primary PRO subscales (i.e. HADS-A, HADS-D) and targeted ROI activity and connectivity for all participants post 6 sessions.

#### Objective 3

Within-group (ISF1 and ISF2) comparisons between post-6 sessions and post-12 sessions will be performed for all outcome measures. The same model will be applied as in Objective 1 where random effects will now allow for post-6 session differences between participants.

#### Objective 4

Within-group (ISF1 and ISF2) comparisons between post-12 sessions and 1-month follow-up will be performed for all outcome measures. This will use the same model as Objective 1 where random effects will now allow for post 12-session differences between participants.

#### Objective 5

Regression analysis from Objective 2 will be extended to post 12-session and 1-month follow-up for the ISF1 and ISF2 groups.

Analyses were performed using LORETA-KEY software, R, JAGS [299] and Stan [300] with analysis-specific details described below. For all endpoints, responses were modelled assuming a normal distribution (e.g. with group-specific means and variances or via regression). When normality assumptions were not met, appropriate transformations were performed. Additionally, potential endpoint covariates were examined (e.g. age); however, none exhibited correlations that were strong enough to warrant inclusion for denoising purposes in the models. For the Bayesian analysis, JAGS or Stan was linked to R using the rjags or rstanarm library with estimates based on 3 chains of 25,000 iterations with a burn-in/warm-up = 10,000 iterations. Vague priors were used throughout.

Bayes’ theorem postulates that the probability of an event A given event B is proportional to the probability of event B given event A multiplied by the prior probability of event A:

$$P\left(A|B\right)\propto P\left(B|A\right)\ast P(A)$$

Bayesian statistical analysis is based on the concept of prior knowledge/beliefs regarding random variables P(A) that are combined with a model relating data to those variables P(B|A) to generate a posterior probability distribution reflecting updated knowledge/beliefs about the variables given the collected data P(A|B) [301].

The describe_posterior() function in the BayestestR package [302] was used to generate posterior summary statistics including the distribution mean (*M*), 95% credible interval (i.e. highest density interval, HDI), probability of direction (pd), and the percentage of the full posterior within the region-of-practical-equivalence (% in ROPE).

- The HDI is the range of parameter values with a higher probability density than values outside the HDI [303]. As such, a 95% HDI can be interpreted as a 95% probability that the true (unknown) estimate lies within the interval, given the observed data and priors [304]. In other words, it is an index of the top 95% most credible parameter values.
- The pd is an index of the *existence* of an effect and is represented by the certainty (50-100%) in the direction, positive or negative [302, 305]. Put simply, it is the percentage of the posterior on the same side as the posterior’s measure of central tendency (e.g. the mean). For pd interpretation, the following reference values have been suggested:
  - ≤95% = uncertain
  - >95% = possibly existing
  - >97% = likely existing
  - >99% = probably existing
  - >99.9% = certainly existing
- The percentage in ROPE indexes the *magnitude* of an effect where the ROPE is the range of effect size values considered to be practically equivalent to the null [302, 305]. There is no uniquely correct ROPE; however, by convention, the ROPE range is often set at half the size of Cohen’s definition of small effect size (i.e. 0.2 [306];) resulting in ROPE values of ±0.1 and ±0.05 for *standardized* mean differences (e.g. Cohen’s *d* = group 1 mean – group 2 mean/pooled standard deviation) and *standardized* regression coefficients (i.e. sβ = coefficient from a regression on standardized variables), respectively [303]. As an aside, Cohen’s *d* values can be interpreted as follows: *d* < ±0.10 = negligible, ±0.10 < *d* < ±0.20 = very small, ±0.20 < *d* < ±0.50 = small, ±0.50 < *d* < ±0.80 = medium, and *d* > ±0.80 = large [306] whereas sβs can be interpreted as follows: sβ < ±0.20 = weak association, ±0.20 < sβ < ±0.50 = moderate association, sβ > ±0.50 = strong association [307]. For percentage in ROPE interpretation, the following reference values have been suggested:
  - >99% = negligible
  - >97.5% = probably negligible
  - ≤97.5 and ≥2.5% = undecided
  - <2.5% = probably significant
  - <1% = significant

For all outputs, checks for the validity of assumptions regarding the residuals (i.e. normal distribution, constant and equal variances), chain convergence (e.g. trace plots), and posterior predictive model fit (e.g. Bayesian *p*-value) will be performed. Of note, a Bayesian *p*-value can be defined as “the probability, given the data, that a future observation is more extreme (as measured by some test variable) than the data” with values near 0.5 indicative of good model fit [308].

### Interim analyses

Not applicable: no interim analyses will be performed, and no stopping guidelines will be established.

### Methods in analysis to handle protocol non-adherence and any statistical methods to handle missing data

We will utilize complete-case analysis for all endpoints and objectives. For post 6-session and/or post 12-session/follow-up data to be included in the analysis, participants must attend a minimum of 5 out of 6 ISF-NFB sessions in each respective 6-session block. Further, we will report the number and percentages of withdrawal in each of the groups. Based on our lab group’s prior feasibility study using ISF-NFB in an obese female population [159], discontinuation/loss-to-follow-up following randomization is expected to be 10-15%.

### Adverse event reporting and harms

We will systematically monitor adverse effects from the therapy for the duration of the trial using the Discontinuation-Emergent Signs and Symptoms checklist (DESS [309]) created, verbatim, in Qualtrics and completed by participants on an iPad during EEG set-ups in the interventional and post-interventional phases. Initially developed for drug trials [309], the DESS is a structured 43-item self-report that utilizes the following scale: 1=new symptom, 2=old symptom but worse, 3=old symptom but improved, 4=old symptom but unchanged, 5=symptom not present. The DESS has been used for the assessment of treatment-related side-effects in ID populations [310, 311] and, recently, has been employed to monitor adverse-effects specifically associated with NFB therapy [312]. Participants may be withdrawn from the trial by the investigators, even without their request, in the event of serious adverse effects. As detailed in the PIS, a brief (4-item) interview used during our group’s prior sLORETA ISF-NFB feasibility trial revealed that, although unusual or vivid dreams were experienced by some participants, there were no serious adverse effects [160].

### Data management and processing

Participant paper files, including case-report-forms (CRFs) and MINI assessments, are to be kept in numerical order and stored in a locked room accessible only to the researchers. PROs will be electronically stored in Qualtrics with a back-up copy automatically generated and sent to the lead researcher’s trial email address. All data collected will be entered into Microsoft Excel (version 2112) and double-checked for accuracy by the data analyst at the time of entry. Participant data will be maintained for a period of not less than 10 years after the completion of the study.

### Confidentiality

All information generated in this study will be considered highly confidential and is not to be shared with any persons not directly concerned with the study. For de-identification purposes, participants will be assigned unique study numbers upon enrolment. All electronic records will be identified solely using assigned study numbers and stored locally in a password-protected database. All paper records will be stored on-site in a locked office accessible only to the researchers directly involved in the trial. Furthermore, paper documents that contain personal identifiers (i.e. informed consent forms), will be stored separately from de-identified paper records (i.e. CRFs and MINIs).

### Access to data

The final trial dataset will be password protected and housed locally at the research lab. Other team members will be provided access to this dataset by TMP upon request. To ensure confidentiality, data dispersed to project team members will be blinded of any identifying participant information.

### Plans for collection, laboratory evaluation and storage of biological specimens for genetic or molecular analysis in this trial/future use

Not applicable: no specimens collected

### Plans to give access to the full protocol, participant level-data and statistical code

The full protocol will be submitted for publication to a peer-reviewed, open-source journal prior to analyses commencement. No more than 2 years following the final data collection, we will deliver the completed, de-identified dataset and statistical code to the appropriate data archive for sharing purposes in line with the scientific imperatives of increased transparency, reproducibility, and interpretation of trials.

### Oversight and monitoring

#### Composition of the coordinating Centre and trial steering committee

Not applicable: no coordinating centre or trial steering committee for this trial

#### Composition of the data monitoring committee, its role and reporting structure

Due to the relatively short duration of recruitment, non-invasive make-up of the procedures/interventions, and non-serious nature of adverse effects reported in our prior feasibility trial, no formal data monitoring committee will be established.

#### Frequency and plans for auditing trial conduct

Not applicable: no auditing of trial conduct will be performed.

#### Plans for communicating important protocol amendments to relevant parties (e.g. trial participants, ethical committees)

Substantive protocol amendments which may impact on the conduct of the study including changes to the study objectives, design, population, sample sizes, or procedures will be agreed upon by the research team, updated in the trial registry, submitted to the ethics committee for approval, and updated on our online trial advertisements and web pages.

#### Dissemination policy

Every effort will be made to minimize the interval between the completion of data collection and release of study results. We estimate this process to take 12 months. Irrespective of magnitude or direction of effect, results from the study will be written up and submitted to international peer-reviewed scientific journals, presented at scientific conferences, and may form part of grant applications. In addition, once compiled, all participants will be provided with a digital copy of the results.

## Discussion

Approximately one in five New Zealanders is dealing with a mental illness at any given time with the majority of the population expected to experience psychopathology at some point in their lifetime [7]. Alarmingly, New Zealand’s suicide numbers are increasing with the 2017–2018 rate the highest it’s been in 20 years [7] contributing to a staggering reduction in life expectancy for mental illness sufferers of up to 25 years [7]. A recent government inquiry by the New Zealand government has shed light on the shortcomings of current treatment and called for wider implementation of non-pharmaceutical approaches in treatment of mental health problems [7]. Similarly, scientists in other parts of the world are calling for research into “novel interventions that may be based on altering plasticity or returning circuitry rather than neurotransmitter pharmacology” [313].

The implementation of safe, non-invasive neuromodulation techniques that have the potential to impact neuroplasticity within and between large-scale ICNs may offer new treatment opportunities for individuals who either do not want, respond to, or tolerate standard interventions. Additionally, these techniques may serve as adjuncts to traditional treatments, potentially enhancing their efficacy. To date, ours is the only research group studying the effects of sLORETA ISF-NFB in clinical populations. We believe targeting core ICNs via this novel therapy offers a promising new avenue in the treatment of IDs and other psychopathologies.

## Trial status

Recruitment began on 15 February 2020 but was prematurely halted due to COVID-19 lockdown measures here in New Zealand. Recruitment efforts resumed on 15 June 2020, however, due to budgetary and time restrictions imposed by the lockdown, we amended our protocol. Specifically, our recruitment goal for clinical participants was changed from 80 (40 males and 40 females) to 60 females. Data collection is on track to be completed by the end of 2021.

## Supplementary Information

**Additional file 1.** MCC voxelsR1.

**Additional file 2.** PCC voxelsR1.

## Abbreviations

aINS: Anterior insula
ANZCTR: Australia New Zealand clinical trials registry
CEN: Central executive network
*d*: Cohen’s *d*
dACC: dorsal Anterior Cingulate Cortex
DESS: Discontinuation-emergent signs and symptoms checklist
dlPFC: Dorsolateral prefrontal cortex
DMN: Default mode network
DSM-5: Diagnostic and statistical manual of mental disorders – 5^th^ Edition
ECG: Electrocardiography
EEG: Electroencephalography
EEG-NFB: Electroencephalographic neurofeedback
eISFs: Electrophysiological infraslow fluctuations
eLORETA: Exact low-resolution electromagnetic tomography
EMG: Electromyography
EOG: Electrooculography
GAD: Generalized anxiety disorder
HADS: Hospital Anxiety and Depression Scale
HRV: Heart rate variability
ICN: Intrinsic connectivity network
ID: Internalizing disorder
IDAS-II: Inventory of Depression and Anxiety Symptoms – Second Version
ISAD: Infraslow closed-loop brain training for Anxiety and Depression
ISF-NFB: Infraslow fluctuation neurofeedback
ISF1: 1-region Infraslow neurofeedback
ISF2: 2-region Infraslow neurofeedback
IUS-12: Intolerance of Uncertainty Scale – 12-item version
MDD: Major depressive disorder
MEDI: Multidimensional Emotional Disorder Inventory
MINI: Mini International Neuropsychiatric Interview
MNI: Montreal Neurological Institute
mPFC: Medial prefrontal cortex
PCC: Posterior cingulate cortex
pd: Probability of direction
PROs: Patient-reported outcomes
ROPE: Region of practical equivalence
RTQ-10: Repetitive Thinking Questionnaire – 10-item trait version
sLORETA: Standardized low-resolution electromagnetic tomography
SN: Salience network
SOC: Social anxiety disorder
